# Constitutive translation of human α-synuclein is mediated by the 5′-untranslated region

**DOI:** 10.1098/rsob.160022

**Published:** 2016-04-20

**Authors:** Pelagia Koukouraki, Epaminondas Doxakis

**Affiliations:** Division of Basic Sciences, Biomedical Research Foundation, Academy of Athens, Athens, Attiki 11527, Greece

**Keywords:** α-synuclein, 5′-UTR, internal ribosome entry site, Parkinson's disease, iron-responsive element, G-quadruplex

## Abstract

Genetic and biochemical studies have established a central role for α-synuclein (SNCA) accumulation in the pathogenesis of Parkinson's disease. Uncovering and subsequently interfering with physiological mechanisms that control SNCA expression is one approach to limit disease progression. To this end, the long and GC-rich 5′-untranslated region (UTR) of SNCA, which is predicted to fold into stable hairpin and G-quadruplex RNA motifs, was investigated for its role in mRNA translation. Inclusion of SNCA 5′-UTR significantly induced expression of both SNCA and luciferase ORF constructs. This effect was not associated with a change in mRNA levels or differential nucleocytoplasmic shuttling. Further, the presence of the 5′-UTR enhanced SNCA synthesis when cap-dependent translation was attenuated with rapamycin treatment. Analysis using multiple methodologies revealed that the 5′-UTR harbours an internal ribosome entry site (IRES) element that spans most of its nucleotide sequence. Signals such as plasma-membrane depolarization, serum starvation and oxidative stress stimulated SNCA protein translation via its 5′-UTR as well as enhanced its IRES activity. Taken together, these data support the idea that the 5′-UTR is an important positive regulator of SNCA synthesis under diverse physiological and pathological conditions, explaining in part the abundance of SNCA in healthy neurons and its accumulation in degenerative cells.

## 1. Background

α-Synuclein (SNCA) is a neuronal protein of as yet poorly characterized function, normally found in presynaptic terminals and likely to be involved in the modulation of synaptic neurotransmission. Converging evidence supports the concept that SNCA pathology is integral to the pathological process in Parkinson's disease (PD) and a number of other diseases now categorized under the term alpha-synucleinopathies. In this regard, point mutations and gene duplication and triplication events in the SNCA locus have been identified in a number of families with autosomal dominant early onset PD [1–3]. Moreover, SNCA is a major component of Lewy bodies (LB) in sporadic PD and dementia with LB, and also of inclusions found in both glial and neuronal cells in multiple system atrophy [4]. *In vitro* cell culture studies indicate that the expression of mutant SNCA can sensitize neurons to toxic challenges, and viral-mediated overexpression of wild-type or mutant SNCA within nigral neurons of rodents and non-human primates has led to progressive motor dysfunction mimicking motor symptoms in PD patients [5–7]. Taken together, these studies suggest that inhibiting SNCA expression may help ameliorate symptoms associated with PD and other synucleinopathies.

Protein expression is regulated at multiple levels and, in particular, at the step of translation initiation. To date, three secondary structural elements that affect translation initiation have been identified in the 5′-untranslated region (UTR) of mRNAs. These are the iron-responsive elements (IREs), the G-quadruplex (G-q) structures and the internal ribosome entry site (IRES) elements. IREs are hairpin structures that attract iron regulatory proteins (IRPs) to normally block the 43S ribosomal recruitment and hence translation [8]. Thus far, an IRE structure has been predicted for the distal segment of the SNCA 5′-UTR but has not been experimentally verified as yet [9]. G-q structures consist of G-quartet planes, which are a planar array of four guanines joined by Hoogsteen hydrogen bonds, with a monovalent cation in the centre. The great majority function as translation repressors that block formation or scanning of the preinitiation complex [10]. IRES elements are typically long, GC-rich and heterogeneous in their sequence. They contain a high degree of RNA secondary structures that recruit the 40S ribosomal subunit in a cap-independent manner. It is predicted that around 5% of all cellular mRNAs use, in addition to cap-dependent translation, this alternative form of translation initiation [11]. Although the mechanism of action is currently not understood, it is thought that some of them require auxiliary factors, called IRES-trans acting factors (ITAFs) to function. IRESs are thought to serve three major physiological functions. First, they support robust translation of mRNAs under conditions that require tight control of expression as in the case of local translation in dendrites [12]. Second, they sustain expression when cap-dependent translation is compromised as in the case of hypoxia, amino acid deprivation, heat-shock and oxidative stress [13–15]. Third, they support low levels of translation initiation of mRNAs with highly structured 5′-UTRs (incompatible with efficient scanning) under normal physiological conditions when cap-dependent translation is active. Not surprisingly, most IRES elements are found in the mRNA of genes involved in the control of cellular proliferation, survival and death, suggesting that IRES-mediated translation plays an important role in the regulation of cell fate by overriding cellular stress signals.

The human *SNCA* mRNA possesses a relatively long 5′-UTR of 264 nt with a high percentage (66%) of GC bases that is predicted to fold into stable secondary structures. Interestingly, several proteins targeted to neuronal synapses have been shown to possess complex 5′-UTRs displaying IRES activity indicating that SNCA, a presynaptic molecular chaperon of SNARE complexes, could exhibit similar properties [12]. These observations led us to examine the role of the SNCA 5′-UTR in overall translation regulation. The analysis revealed that the SNCA 5′-UTR significantly promotes cap-dependent translation and that IRES-mediated translation is a mechanism by which endogenous *SNCA* mRNA may be translated. Moreover, conditions of stress such as serum starvation, increased intracellular iron accumulation and depolarization augmented both SNCA IRES activity and 5′-UTR-mediated enhanced SNCA protein synthesis. These results indicate that the SNCA 5′-UTR is an important mediator of SNCA protein levels and maybe a relevant therapeutic target for modulating SNCA expression in alpha-synucleinopathies.

## 2. Results

### 2.1. The SNCA 5′-UTR regulates SNCA protein expression

**Figure 1.**
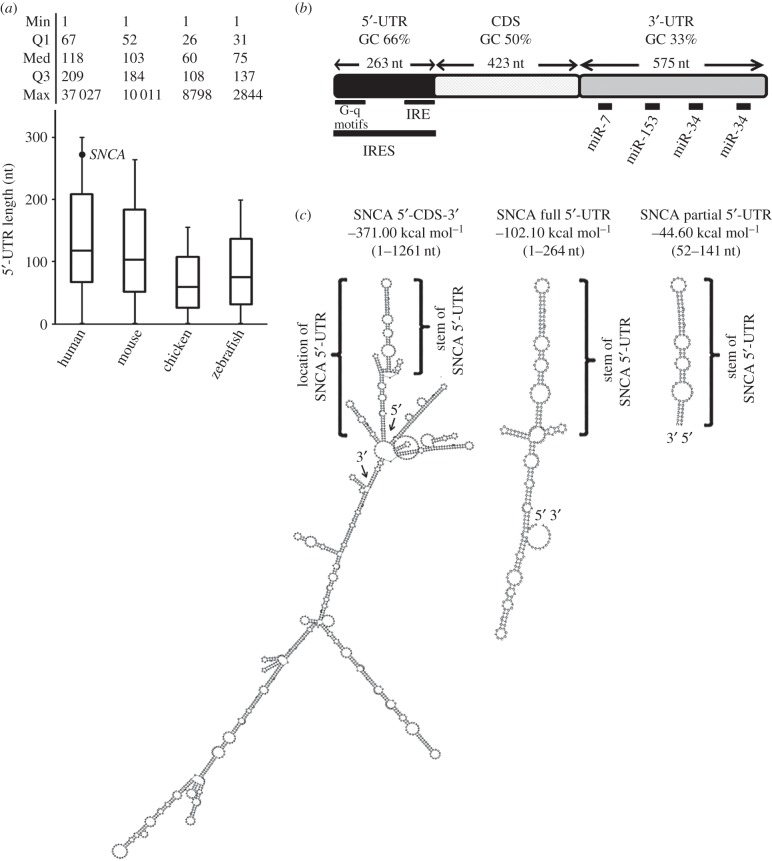
Bioinformatics analysis of SNCA 5′-UTR. (*a*) Box plot analysis of 5′-UTR lengths of human, mouse, chicken and zebrafish genes according to RefSeq dataset. Human SNCA 5′-UTR (NM_000345.3) length is depicted as a single dot. (*b*) Schematic of human *SNCA* mRNA transcript. GC content, nucleotide length and regulatory elements validated and/or predicted are indicated. (*c*) Secondary structure predictions of the *SNCA* mRNA according to RNAfold (ViennaRNA package 2.0). The 5′ and 3′ ends of *SNCA* mRNA as well as the 5′-UTR region are indicated. Note that folding of SNCA 5′-UTR is preserved irrespective of the length of nucleotides used in the RNAfold analysis.

**Figure 2.**
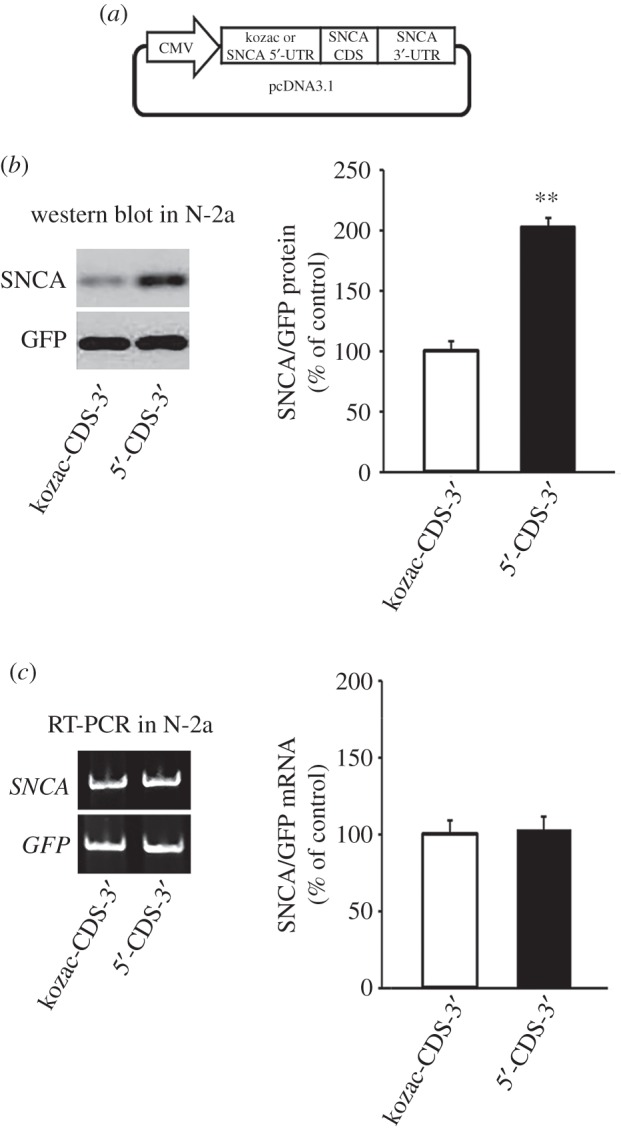
The SNCA 5′-UTR enhances SNCA open reading frame (ORF) translation. (*a*) Schematic of the SNCA pcDNA3.1 monocistronic expression constructs. The SNCA cDNA with (5′-CDS-3′) or without (kozac-CDS-3′, control), the 5′-UTR was inserted downstream of the CMV promoter in the pcDNA3.1 expression plasmid. (*b,c*) SNCA pcDNA3.1 plasmids were transiently co-transfected into Neuro-2a (N-2a) cells with Emerald GFP (EmGFP)-DNA6.2 plasmid serving as an internal control of transfection (the scramble miR sequence at EmGFP 3′-UTR in the original vector was removed by double digestion). Forty-eight hours later, cells were harvested and SNCA protein and mRNA levels were determined and normalized to EmGFP levels following immunoblotting and real-time RT-PCR, respectively. Note that the SNCA 5′-UTR induced protein but not mRNA levels. The figures represent averages and standard errors of four to six independent experiments. **p < 0.01.

The median human 5′-UTR length, similar to other vertebrate species, is around 120 nt (figure 1*a*). By contrast, the 5′-UTR of human *SNCA* mRNA (NM_000345.3) is more than twice as long, composed of 264 nt, and has a high GC content of 66% indicating that it is likely to fold into elaborate RNA secondary structures to regulate overall protein output (figure 1*b*). To test this possibility, the RNAfold algorithm (ViennaRNA package 2.0) [16] was used to predict folding of both total *SNCA* mRNA and its 5′-UTR alone. Analysis confirmed that the 5′-UTR forms secondary structures that are largely preserved irrespective of the length of *SNCA* mRNA nucleotides used in the enquiry (figure 1*c* and data not shown). The minimum free energy of folding for the SNCA 5′-UTR was predicted at −102 kcal mol^−1^ while the 5′-UTR hairpin formed by nt 52–141 was estimated to have a Δ*G* value of −46 kcal mol^−1^ (figure 1*c*). Given that RNA stem-loop structures with a free energy lower than −30 kcal mol^−1^ have been shown to be inhibitory to cap-dependent ribosomal scanning [17,18], it was predicted that the SNCA 5′-UTR will attenuate cap-dependent translation. To test this, two kinds of pcDNA3.1 expression constructs were prepared: the 5‘-CDS-3’ construct that contained coding and both 5′- and 3′-UTR regions and the kozac-CDS-3’ construct that contained the coding with the kozac consensus nucleotide sequence upstream of ATG (cgccgccATG) and the 3′-UTR region of human *SNCA* gene (figure 2*a*; electronic supplementary material, table S1 for primer sequences). These SNCA expression constructs were then transiently transfected into low-passage murine neuroblastoma Neuro-2a cells, that express barely detectable endogenous SNCA protein, and immunoblotting for SNCA protein levels was conducted. Unexpectedly, the protein levels of SNCA produced by the expression construct that included the 5′-UTR (5′-CDS-3′) were found to be over 100% higher than those of the kozac-CDS-3′ construct (Student's *t*-test, **p < 0.01; figure 2*a*). At the same time, the levels of *SNCA* mRNA produced by both constructs were the same, indicating that the disparity observed in the protein levels was due to differential regulation at the translation level (figure 2*b*).

**Figure 3.**
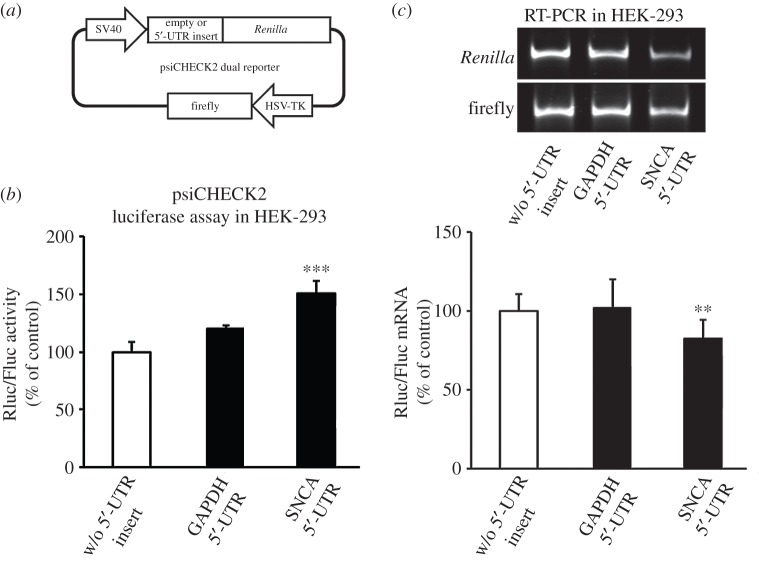
The SNCA 5′-UTR enhances luciferase reporter activity. (*a*) Schematic of the psiCHECK2 bicistronic expression constructs. The SNCA and GAPDH 5′-UTR cDNAs were inserted upstream of *Renilla* luciferase gene in the psiCHECK2 dual reporter vector. (*b,c*) Empty (no insert) control plasmid and plasmids containing the SNCA or GAPDH 5′-UTR were transiently transfected into HEK-293 cells. Forty-eight hours later, cells were harvested and dual luciferase assays was performed by sequentially measuring the firefly and *Renilla* luciferase activities of the same sample, with the results expressed as the ratio of *Renilla* to firefly (Rluc/Fluc) activity. Relative mRNA levels were determined by real-time RT-PCR. The results are expressed as the ratio of *Renilla* to firefly expression. Note that only the psiCHECK2 construct that incorporates the SNCA 5′-UTR induced luciferase protein levels. The figures represent averages and standard errors of four independent experiments. *p < 0.05, ***p < 0.001.

To further address the role of the SNCA 5′-UTR in translation regulation, the SNCA and GAPDH 5′-UTRs were next cloned ahead of the *Renilla* luciferase gene in the psiCHECK2 dual reporter vector (figure 3*a*). Easy-to-transfect HEK-293 cells were then transiently transfected with these plasmids and dual reporter and real-time RT-PCR assays were performed 48 h later. Similar to previous results, inclusion of SNCA, but not GAPDH, 5′-UTR increased Renilla luciferase levels by over 50% compared with empty control (one-way ANOVA, *F*_2,17_ = 16.917, *p* < 0.001 and LSD *post hoc*; figure 3*b*). Importantly, the levels of *Renilla* mRNA produced by the SNCA 5′-UTR reporter construct were not higher, but in fact some 20% lower than empty and GAPDH controls (one-way ANOVA, *F*_2,20_ = 8.399, *p* < 0.01; figure 3*c*). Taken together, these data suggested that the 5′-UTR of *SNCA* mRNA possesses element(s) that significantly enhance translation.

Cryptic AUGs and secondary structure elements within 5′-UTRs are known to modify translation initiation of mRNAs and hence protein output. With respect to *SNCA* mRNA, no AUG triplet is present on the 5′-UTR and no alternative initiation has, so far, been reported. Currently, three secondary structure elements that affect translation initiation have been identified. These are the IREs, the G-q structures and the IRESs. Thus far, an IRE structure has been predicted just before the start codon (nt 218–263) but has not been experimentally verified as yet [9]. To test if the RNA binding proteins (RBPs) IRP-1 and IRP-2 are involved in the SNCA 5′-UTR-mediated translation, RNA pull-down assay of total murine postnatal day 3 brain extracts using biotin-labelled SNCA 5′-UTR mRNA as bait, followed by nano combination of liquid chromatography with mass spectrometry (LC-MS/MS) proteomics for the detection of all interacting proteins was carried out. Among the hundreds of interactors identified, neither IRP-1 nor IRP-2 proteins were pulled down (data not shown). Similarly, other studies using RNA immunoprecipitation (RIP) analysis with IRP-1/2 antibodies in brain and bone-marrow tissues, both highly enriched in *SNCA* mRNA, did not detect *SNCA* mRNA among the targets [19]. Based on these findings, no further analysis with respect to predicted IRE element was conducted.

**Figure 4.**
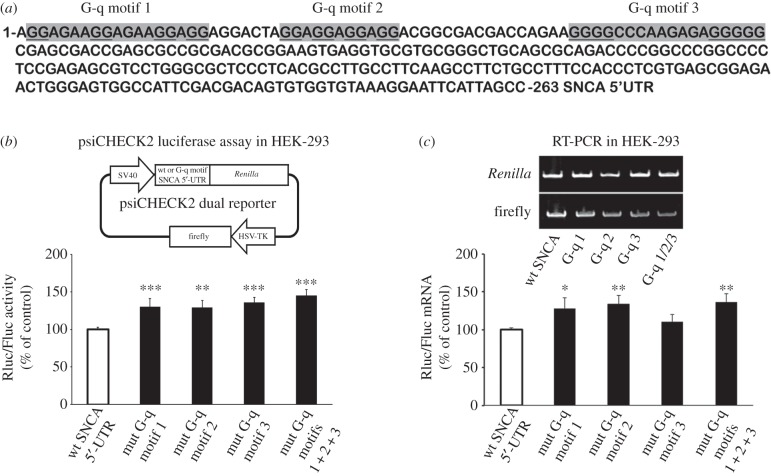
The G-quadruplex motifs located on proximal SNCA 5′-UTR inhibit luciferase reporter activity. (*a*) Schematic of the three non-overlapping G-quadruplex motifs predicted for SNCA 5′-UTR. (*b,c*) SNCA 5′-UTR reporter psiCHECK2 plasmids with wild-type (control) or mutagenized G-quadruplex motifs were transiently transfected into HEK-293 cells. Forty-eight hours later, cells were harvested and dual luciferase assays were performed by sequentially measuring the firefly and *Renilla* luciferase activities of the same sample, with the results expressed as the ratio of *Renilla* to firefly (Rluc/Fluc) activity. Relative mRNA levels were determined by real-time RT-PCR. The results are expressed as the ratio of *Renilla* to firefly expression. Note that the SNCA 5′-UTR displays relatively weak G-quadruplex formations. The figures represent averages and standard errors of four independent experiments. *p < 0.05, **p < 0.01, ***p < 0.001.

G-q structures in mRNA molecules have recently emerged as important regulators of translation. To test if they are involved in SNCA translation, the G-q secondary prediction algorithm QGRS Mapper was employed [20] and three non-overlapping motifs were detected at the proximal SNCA 5′-UTR (figure 4*a*). Their predicted G-scores were rather mediocre compared to QGRS scores from genes with verified G-q motifs (electronic supplementary material, table S2) [21–24]. Nevertheless, four psiCHECK2 reporter constructs were prepared in which each of the predicted G-q motifs or all together were mutagenized. Transient transfection of HEK-293 cells with these constructs revealed that the activity of *Renilla* luciferase increased 30%, 29%, 36% and 45% by mutations in G-q motifs 1, 2, 3 and 1/2/3, respectively (one-way ANOVA, *F*_4,15_ = 9.981, *p* < 0.001 and LSD *post hoc*; figure 4*b*). The levels of *Renilla* mRNA produced by the mutant constructs, except G-q motif 3 were approximately 30% higher than that of wild-type SNCA 5′-UTR control construct (one-way ANOVA, *F*_4,20_ = 3.903, *p* < 0.05 and LSD *post hoc*; figure 4*c*). Therefore, with the exception of G-q motif 3 mutation that significantly enhanced translation initiation of *SNCA* mRNA without altering mRNA levels, the mutations on predicted G-q motifs 1 and 2 promoted translation by either enhancing transcription or stabilizing SNCA 5′-UTR reporter mRNA. Taken together, these predicted G-q motifs, if preserved on native *SNCA* mRNA, are likely to mediate a negative response on *SNCA* mRNA translation.

### 2.2. The SNCA 5′-UTR does not mediate nuclear export

**Figure 5.**
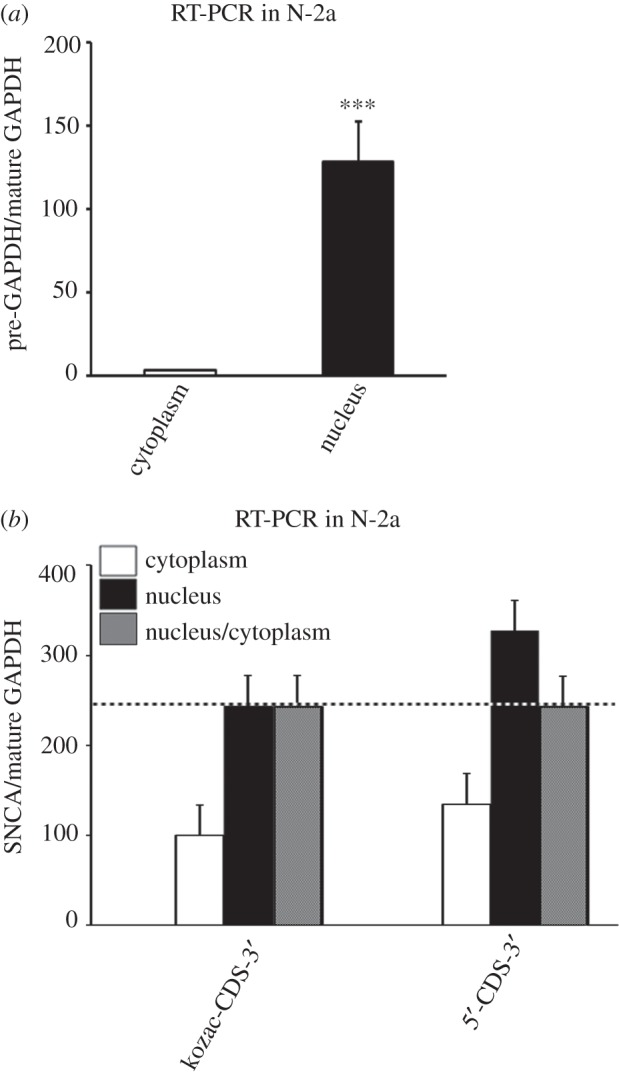
The SNCA 5′-UTR does not mediate nuclear export. (*a*) Neuro-2a cells were fractionated into nuclear and cytoplasmic fractions and pre- and mature *GAPDH* mRNA levels were determined by real-time RT-PCR. Note that *GAPDH* pre-mRNA is primarily detected in the nuclear fraction. (*b*) The SNCA pcDNA3.1 5′-CDS-3′ and kozac-CDS-3′ plasmids were transiently transfected into Neuro-2a cells. Forty-eight hours later, cells were harvested, fractionated into nuclear and cytoplasmic fractions and *SNCA* and mature *GAPDH* mRNA levels were determined by real-time RT-PCR. Note that SNCA 5′-CDS-3′ and kozac-CDS-3′ are exported to cytoplasm at the same rates. The figure represents averages and standard errors of three independent experiments. ***p < 0.001.

An obvious mechanism by which SNCA 5′-UTR could mediate much of its effects on cap-dependent translation is via enhanced nuclear export. To test this, Neuro-2a cells transfected with either the pcDNA3.1 5′-CDS-3′ or the kozac-CDS-3′ construct were subjected to subcellular fractionation and analysed by real-time RT-PCR. Figure 5*a* shows that *GAPDH* pre-mRNA was 50-fold enriched in the nuclear fraction indicating an efficient fractionation method (Student's *t*-test, ***p < 0.001). Figure 5*b* shows that the distributions of 5′-CDS-3′ and kozac-CDS-3′ mRNAs in the nucleus and cytoplasm were the same (one-way ANOVA, *F*_3,8_ = 1.478, *p* = 0.292 and LSD *post hoc*). Overall, these data indicate that the SNCA 5′-UTR does not mediate nuclear export, strengthening the assumption that the SNCA 5′-UTR is specifically involved in translation initiation in the cytoplasm.

### 2.3. The SNCA 5′-UTR enhances translation when cap-dependent ribosomal scanning is inhibited

**Figure 6.**
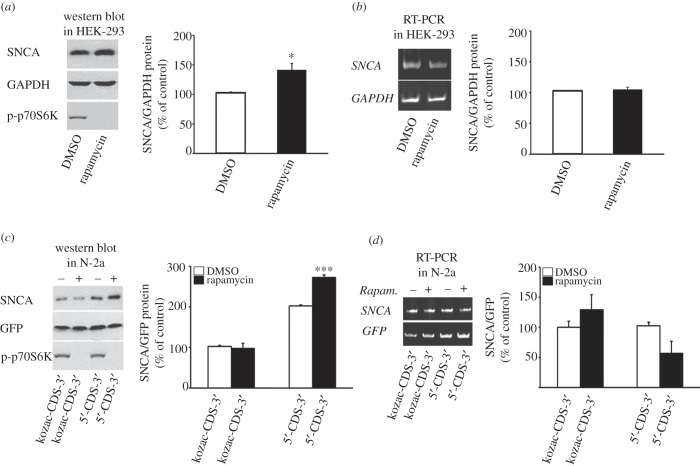
The SNCA 5′-UTR mediates cap-independent translation of *SNCA* mRNA. Freshly plated HEK-293 cells (*a,b*) were treated with either DMSO or translation blocker rapamycin. Forty-eight hours later, cells were harvested and SNCA protein and mRNA levels were determined and normalized to GAPDH protein levels following immunoblotting. The phosphorylated levels of p70S6K, a substrate of mTORC1, were also assayed to confirm the effectiveness of drug treatment. *SNCA* mRNA levels were determined and normalized to *GAPDH* mRNA levels following real-time RT-PCR. Note that rapamycin treatment induced SNCA protein but not mRNA levels. (*c,d*) Monocistronic pcDNA3.1 plasmids expressing SNCA ORF with or without SNCA 5′-UTR were transiently co-transfected into Neuro-2a cells with EmGFP plasmid serving as an internal control of transfection. Three hours later, they were treated with either DMSO or rapamycin to attenuate cap-dependent translation. Forty-eight hours post-transfection, cells were harvested and SNCA protein was determined and normalized to EmGFP protein levels following immunoblotting. The phosphorylated levels of p70S6K confirmed the effectiveness of rapamycin treatment. *SNCA* mRNA levels were determined and normalized to *EmGFP* mRNA levels following real-time RT-PCR. Note that rapamycin induced SNCA protein, but not mRNA, expression only from the plasmid construct that contained SNCA 5′-UTR. The figures represent averages and standard errors of four independent experiments. *p < 0.05, ***p < 0.001.

IRESs allow cap-independent translation by directly landing the ribosomal subunit close to the initiator AUG. To determine if SNCA synthesis could be initiated by a cap-independent mechanism, HEK-293 cells, that expressed high levels of SNCA, were treated for 48 h with the mTORC1 translation inhibitor rapamycin. To verify efficient inhibition of cap-dependent translation, the phosphorylated levels of mTORC1 substrate p70S6K were also evaluated. Figure 6*a* shows that in the presence of rapamycin, the protein levels of SNCA were increased by 41% compared with GAPDH control (Student's *t*-test, *p < 0.05). Meanwhile, the levels of phosphorylated p70S6K effector were significantly diminished indicating an effective inhibition of mTORC1 signalling by this drug treatment. Further, real-time RT-PCR analysis of endogenous *SNCA* mRNA levels showed no significant difference between untreated and rapamycin-treated HEK-293 cells, indicating that the change in SNCA protein expression was due to differential regulation of translation (Student's *t*-test, *p* = 0.21; figure 6*b*). SNCA has a long half-life (approx. 47 h) [25] and to exclude interference from endogenous SNCA protein already expressed in cells at the time of drug treatment, this experiment was also contacted in Neuro-2a cells that were transiently transfected with the SNCA 5′-CDS-3′ or kozac-CDS-3′ expression plasmids and treated with rapamycin 4 h later. While rapamycin treatment did not affect SNCA protein levels produced by the kozac-CDS-3′ plasmid, it enhanced levels from the 5′-CDS-3′ plasmid by over 30% (one-way ANOVA, *F*_3,12_ = 24.144, *p* < 0.001 and LSD *post hoc*) indicating that *SNCA* mRNA bearing its 5′-UTR continued to be translated when cap-dependent initiation was attenuated (figure 6*c*). The increase in protein translation of *SNCA* mRNA mediated by its 5′-UTR by means of cap-independent translation is presumed to be the result of increased ribosomal and/or eukaryotic initiation factors (eIFs) availability for alternative translation initiation as previously reported [26–28]. Further, real-time RT-PCR analysis of *SNCA* mRNA levels produced by both plasmids showed no significant difference between untreated and rapamycin-treated conditions, indicating that the change in SNCA protein expression observed was due to differential regulation of translation (one-way ANOVA, *F*_3,12_ = 2.235, *p* = 0.137 and LSD *post hoc*; figure 6*d*). Taken together, these data indicate that the SNCA 5′-UTR mediates cap-independent translation and suggests that it may house an IRES element.

### 2.4. The SNCA 5′-UTR exhibits IRES activity

**Figure 7.**
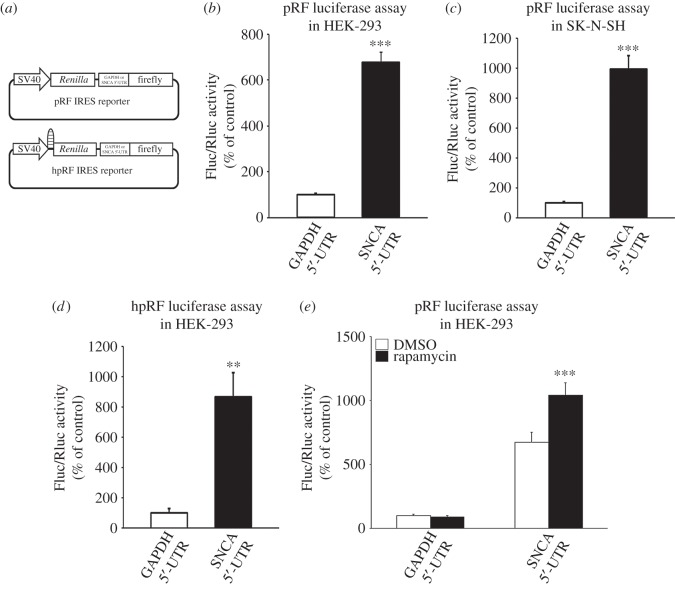
The SNCA 5′-UTR harbours an internal ribosomal entry site (IRES) element. (*a*) Schematic of the pRF and hpRF bicistronic expression constructs. hpRF contains a palindromic sequence upstream of the *Renilla* gene that allows the formation of a stable hairpin in the mRNA known to reduce cap-dependent translation. The SNCA or GAPDH 5′-UTRs were inserted in the intercistronic region of pRF and hpRF plasmids. (*b,c*) HEK-293 and SK-N-SH cells were transiently transfected with SNCA or GAPDH pRF plasmids; (*d*) HEK-293 cells were transfected with SNCA or GAPDH hpRF plasmids. Forty-eight hours later, cells were harvested and dual luciferase assays were performed by sequentially measuring the firefly and *Renilla* luciferase activities of the same sample, with the results expressed as the ratio of firefly to *Renilla* (Fluc/Rluc) activity. Note that SNCA 5′-UTR induced firefly expression. (*e*) HEK-293 cells were transiently transfected with SNCA or GAPDH pRF plasmids. Three hours later, cells were treated with either DMSO or translation blocker rapamycin. Forty-eight hours later, cells were harvested and dual luciferase assays were performed by sequentially measuring the firefly and *Renilla* luciferase activities of the same sample, with the results expressed as the ratio of firefly to *Renilla* (Fluc/Rluc) activity. Note that SNCA IRES activity became more prominent when cap-dependent translation was attenuated. The figures represent averages and standard errors of four independent experiments. **p < 0.01, ***p < 0.001.

To test if the 5′-UTR of *SNCA* mRNA exhibits IRES activity, the bicistronic pRF reporter constructs were used (kindly provided by Dr Hengst L) (figure 7*a*) [29,30]. The SNCA 5′-UTR was cloned between the cDNAs encoding *Renilla* and firefly cistrons in these constructs. These plasmids were then transiently transfected into HEK-293 and SK-N-SH cells and the activity of both luciferases was determined 48 h later. As predicted, the presence of the SNCA 5′-UTR stimulated expression of the downstream cistron 6.8- and 9.9-fold higher than the GAPDH 5′-UTR (control) in HEK-293 and SK-N-SH cells, respectively (Student's *t*-test, ***p < 0.001 for both; figure 7*b,c*). To ensure that internal ribosome entry rather than enhanced ribosomal readthrough, leaky scanning or jumping was responsible for the stimulation observed, (i) the hpRF reporter construct and (ii) rapamycin treatment of pRF transfected HEK-293 cells were employed. The hpRF construct harbours a palindromic sequence upstream of the *Renilla* gene that when transcribed forms a stable RNA hairpin (−55 kcal mol^−1^) [29]. Under this circumstance, the cap-dependent translation of the upstream *Renilla* cistron is diminished, whereas cap-independent IRES activity of the downstream firefly cistron is not affected. HEK-293 cells were chosen for subsequent experiments because they exhibit higher transfection efficiencies. Analysis revealed that the expression of downstream firefly luciferase mediated by the SNCA 5′-UTR was 8.7-fold higher than the one mediated by the GAPDH 5′-UTR (Student's *t*-test, **p < 0.01; figure 7*d*). This suggests that translation of the second cistron is specifically driven by the SNCA 5′-UTR and is not dependent on ribosomal scanning from cap-dependent translation. Similarly, when the pRF constructs were incubated with the cap-dependent translation inhibitor rapamycin an 11-fold increase in downstream firefly luciferase was observed with the SNCA 5′-UTR compared with the GAPDH 5′-UTR control in HEK-293 cells (one-way ANOVA, *F*_3,18_ = 72.125, *p* < 0.001 and LSD *post hoc*; figure 7*e*). These data further demonstrated that translation of the second cistron is mediated by the SNCA 5′-UTR in a cap-independent manner.

**Figure 8.**
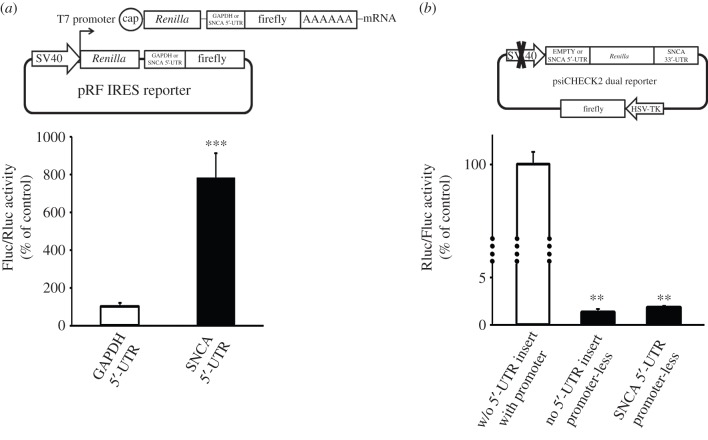
The SNCA 5′-UTR displays no cryptic promoter activity. (*a*) The SNCA and GAPDH pRF bicistronic expression constructs were *in vitro* transcribed from the T7 promoter located upstream of the *Renilla* gene. The mRNAs were then purified free of plasmids and transiently transfected into HEK-293 cells. Seven hours later, cells were harvested and dual luciferase assays were performed by sequentially measuring the firefly and *Renilla* luciferase activities of the same sample, with the results expressed as the ratio of firefly to *Renilla* (Fluc/Rluc) activity. Note that inclusion of SNCA 5′-UTR induced firefly expression. (*b*) HEK-293 cells were transiently transfected with promotor-less bicistronic psiCHECK2 expression constructs containing no 5′-UTR or SNCA 5′-UTR ahead of the *Renilla* gene. Forty-eight hours later, cells were harvested and dual luciferase assays were performed by sequentially measuring the firefly and *Renilla* luciferase activities of the same sample, with the results expressed as the ratio of *Renilla* to firefly (Rluc/Fluc) activity. Note that SNCA 5′-UTR did not possess cryptic promoter activity. The figures represent averages and standard errors of four independent experiments. **p < 0.01, ***p < 0.001.

To exclude the possibility that the translation of the second (firefly) cistron on pRF is derived from a monocistronic mRNA, the transcription of which is initiated by a cryptic promoter present in the SNCA 5′-UTR, the GAPDH and SNCA pRF reporter constructs were *in vitro* transcribed via the T7 promoter at the start of the *Renilla* gene (figure 8*a*). HEK-293 cells were transfected with these RNAs and 7 h later the cell lysates were harvested and assayed for dual luciferase activity. Similar to the results obtained with the DNA constructs, inclusion of the SNCA 5′-UTR ahead of the second cistron increased luciferase activity by 8-fold compared with the GAPDH 5′-UTR control (Student's *t*-test, ***p < 0.001). To further exclude the presence of a cryptic promoter in the SNCA 5′-UTR, the SV40 promoter in the empty and SNCA 5′-UTR psiCHECK2 reporters was removed and the activity of both luciferases was determined, as before. As predicted from previous findings, the SNCA 5′-UTR did not exhibit promoter activity. The leaky *Renilla* activity of both empty and SNCA 5′-UTR promoter-less constructs was estimated to be 80-fold lower than the activity produced by the intact SV40 psiCHECK2 plasmid (one-way ANOVA, *F*_2,8_ = 4026.190, *p* < 0.001 and LSD *post hoc*; figure 8*b*). These results further demonstrate that the SNCA 5′-UTR is devoid of cryptic promoter activity.

**Figure 9.**
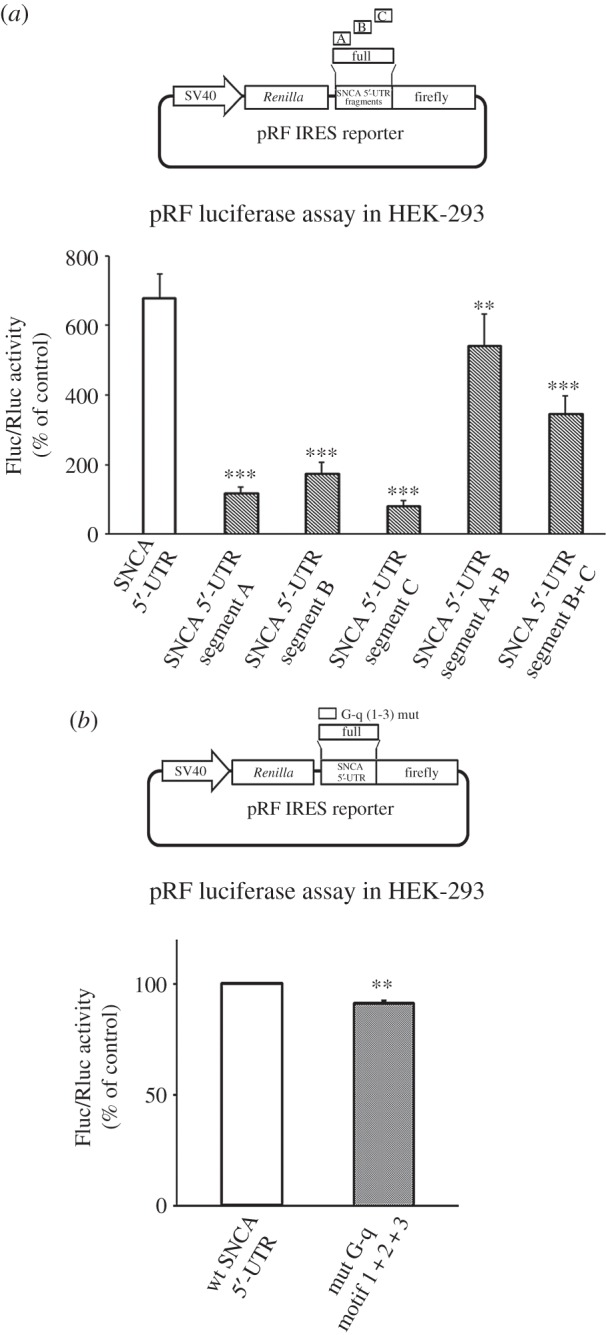
The full-length SNCA 5′-UTR is required for IRES activity. (*a*) The SNCA 5′-UTR was divided into three non-overlapping regions of approximately 88 nt. The three SNCA segments were then inserted into the intercistronic region of pRF plasmid. Note that all segments are required for optimal SNCA IRES activity. (*b*) The three predicted G-quadruplex motifs (located in segment A) were mutagenized in the SNCA pRF plasmid. Control and mutagenized plasmids were, then, transiently transfected into HEK-293 cells. Forty-eight hours later, cells were harvested and dual luciferase assays were performed by sequentially measuring the firefly and *Renilla* luciferase activities of the same sample, with the results expressed as the ratio of firefly to *Renilla* (Fluc/Rluc) activity. Note that the G-quadruplex motifs were dispensable for SNCA IRES activity. The figures represent averages and standard errors of four independent experiments. **p < 0.01, ***p < 0.001.

To locate the region of SNCA 5′-UTR that harbours the IRES element, the 264 nt 5′-UTR sequence was artificially divided into three non-overlapping segments of approximately 85 nt. These fragments were amplified from the original pRF SNCA 5′-UTR reporter and inserted into the empty pRF plasmid. Transfection of these constructs in HEK-293 cells showed that all displayed significantly lower activity than intact 5′-UTR. Some activity was retained with the middle segment (26% of the full-length 5′-UTR) while segments A (17% of full length) and C (12% of full length) displayed very little activity (one-way ANOVA, *F*_5,16_ = 42.711, *p* < 0.001; figure 9*a*). When fragments A + B and B + C were put together, 80% and 50% of the IRES activity was recovered, respectively, but the values were still significantly lower than those obtained from intact 5′-UTR. Additionally, a pRF SNCA 5′-UTR reporter was constructed in which all three predicted G-q motifs (located in segment A) were mutagenized. Results from luciferase experiments showed a significant but not dramatic decrease in IRES activity (Student's *t*-test, **p < 0.01; figure 9*b*). Overall, these data indicated that (i) the middle part of the SNCA 5′-UTR is the most critical for activity, however the full length is required for optimal IRES activity, and (ii) the predicted G-q motifs are not participating in IRES translation initiation. Similar findings have been obtained from the segmentation analysis of other IRES genes [31,32].

### 2.5. The SNCA 5′-UTR enhances translation during different kinds of stress

**Figure 10.**
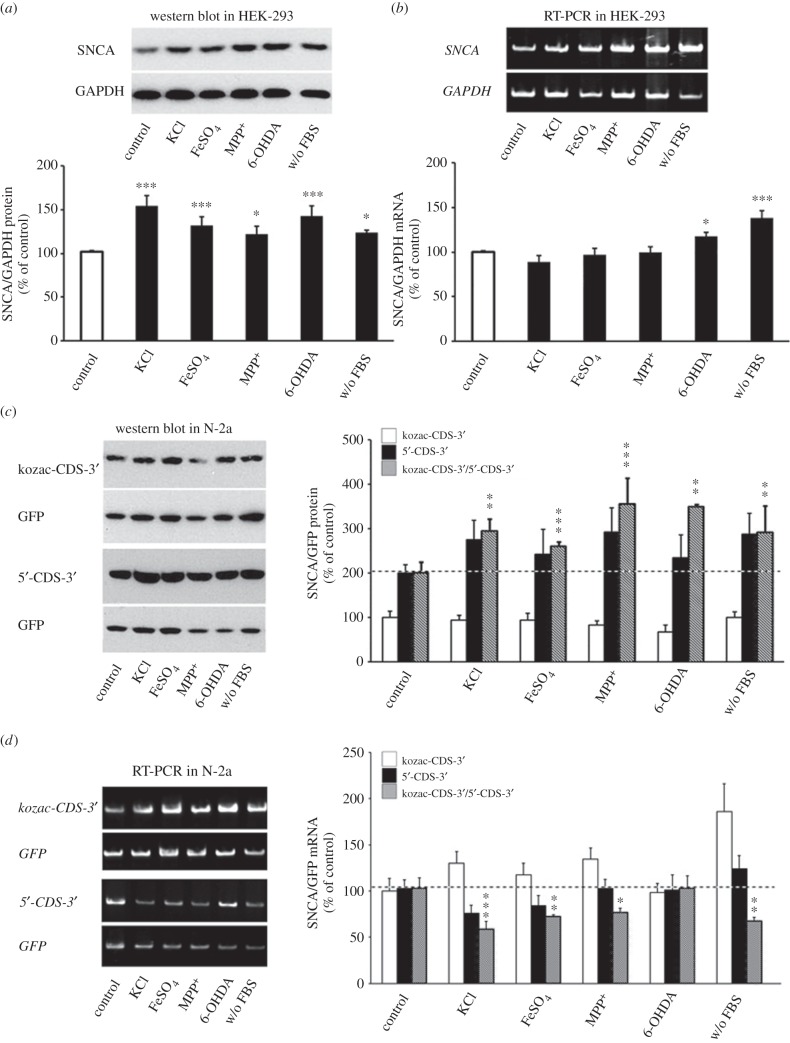
The SNCA 5′-UTR enhances SNCA ORF translation during stress. Freshly plated HEK-293 cells were either serum-deprived (w/o FBS) or treated with different stressors (KCl, ferrous ammonium, 6-OHDA and MPP^+^). Forty-eight hours later, cells were harvested and SNCA protein (*a*) and mRNA (*b*) levels were determined and normalized to GAPDH following immunoblotting and real-time RT-PCR, respectively. Note that all treatments induced SNCA protein levels. From these, 6-OHDA and serum deprivation also induced *SNCA* mRNA levels indicating that their effect on SNCA protein levels may, in part, be mediated by enhanced mRNA expression. (*c,d*) The SNCA pcDNA3.1 5′-CDS-3′ and kozac-CDS-3′ plasmids were transiently co-transfected into Neuro-2a cells with DNA6.2-GW/EmGFP plasmid which served as an internal control of transfection. Four hours later, cells were treated with different stress stimuli. Forty-eight hours later, cells were harvested and SNCA protein and mRNA levels were determined and normalized to EmGFP levels following immunoblotting and real-time RT-PCR, respectively. Note that SNCA 5′-UTR promoted translation under different stress conditions. The figures represent averages and standard errors of four to six independent experiments. *p < 0.05, **p < 0.01, ***p < 0.001.

SNCA expression is deregulated in PD and after treatment with various stress inducers both *in vitro* and *in vivo* [33–35]. Therefore, we next analysed whether the SNCA 5′-UTR mediates any of these effects. Initially, HEK-293 cells received different stress treatments for 48 h and SNCA protein and mRNA levels were analysed by immunoblotting and real-time RT-PCR, respectively. Figure 10*a* shows that depolarization with KCl, iron accumulation, serum deprivation and oxidative stress with ferrous ammonium, 6-OHDA and MPP^+^ all induced significant increase in SNCA protein levels (one-way ANOVA, *F*_5,19_ = 9.912, *p* < 0.001). At the same time, analysis of *SNCA* mRNA levels revealed significant upregulation in 6-OHDA and the serum deprivation treatment conditions (one-way ANOVA, *F*_5,18_ = 16.584, *p* < 0.001; figure 10*b*). These data indicated that depolarization and oxidative stress with ferrous ammonium and MPP^+^ promote translation of *SNCA* mRNA. To specifically test if these effects were mediated by the SNCA 5′-UTR, Neuro-2a cells, transfected with either the pcDNA3.1 5′-CDS-3′ or the kozac-CDS-3′ construct, were treated with the same substances for 48 h and SNCA protein and mRNA levels were analysed by immunoblotting and real-time RT-PCR, as before. Figure 10*c* shows that depolarization with KCl, iron accumulation with ferrous ammonium, oxidative stress induced by 6-OHDA or MPP^+^ and serum deprivation all significantly induced SNCA protein levels when the SNCA 5′-UTR was ahead of the coding region (one-way ANOVA, *F*_6,21_ = 6.403, *p* = 0.001). Interestingly, the opposite effect was reflected in the mRNA levels (one-way ANOVA, *F*_5,21_ = 4.46, *p* < 0.01; figure 10*d*). Taken together, these data show that the SNCA 5′-UTR is a positive mediator of translation, but not of mRNA stability/expression, during cellular stress.

**Figure 11.**
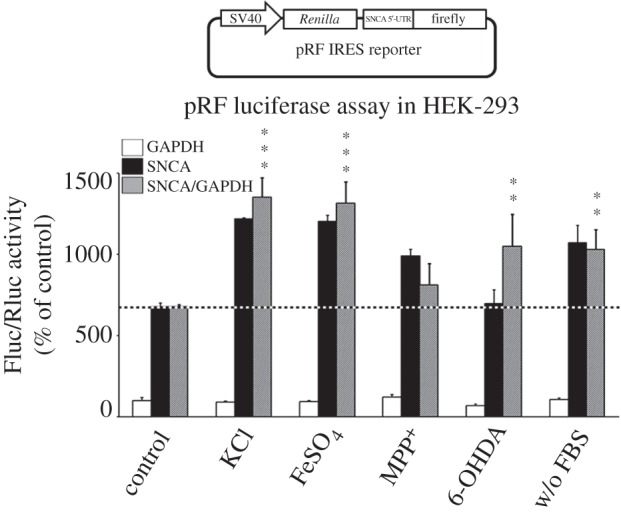
The SNCA IRES activity is induced by different stress signals. HEK-293 cells were transiently transfected with SNCA or GAPDH pRF plasmids. Four hours later, cells were treated with different stress stimuli. Forty-eight hours later, cells were harvested and dual luciferase assays were performed by sequentially measuring the firefly and *Renilla* luciferase activities of the same sample, with the results expressed as the ratio of firefly to *Renilla* (Fluc/Rluc) activity. Note that KCl, FeSO_4_, 6-OHDA, serum deprivation but not MPP^+^ treatment induced SNCA IRES activity. The figure represents averages and standard errors of four independent experiments. **p < 0.01, ***p < 0.001.

IRES-dependent translation is thought to gain importance during stress, when cap-dependent translation is attenuated. To test if any of the above treatments alter SNCA IRES activity, HEK-293 cells were transfected with the pRF GAPDH and SNCA constructs and 4 h later treated with the different substances for approximately 48 h. The cell lysates were subsequently harvested and assayed for luciferase activity as before. Figure 11 shows that KCl, iron, 6-OHDA and serum deprivation, but not MPP^+^, significantly induced SNCA IRES activity, indicating that internal initiation contributes to SNCA accumulation in these conditions (one-way ANOVA, *F*_5,18_ = 6.644, *p* = 0.001).

## 3. Discussion

There are two major findings in this study. First, the SNCA 5′-UTR significantly promotes translation under physiological and stress conditions despite the existence of distinct features (increased length, high GC content, predicted G-q motifs and stable hairpin conformations) presumed to inhibit cap-dependent initiation. Second, the SNCA 5′-UTR harbours an IRES element to sustain SNCA synthesis under unfavourable growth conditions or presumably when controlled translation via ITAFs is required.

The evidence for an inhibitory effect of stable RNA secondary structures is clear from original experiments using artificial sequences of varying GC content forming hairpins of defined stability and placement within the 5′-UTR. In these experiments, hairpins close to the start of the 5′-UTR or hairpins rich in GCs nearly completely abolished translation initiation [17,18,36]. These results were predicted as (i) GC base pairs are more energetically favourable than AU base pairs, allowing the formation of stable secondary structures, and (ii) the 43S preinitiation complex only accommodates single-stranded mRNA in its decoding site. It was, therefore, intriguing to find that inclusion of the long and proximally GC-rich SNCA 5′-UTR mediated cap-dependent translation more efficiently than SNCA or luciferase ORF alone and in levels higher than those produced by inclusion of the 5′-UTR from highly expressed genes such as GAPDH. Current knowledge of what makes a 5′-UTR amenable to cap-dependent translation is insufficient to explain these findings, but one could imagine the presence of yet unidentified enhancer 5′-UTR elements or the action of specialized RBPs.

There is currently concrete evidence of three elements identified in 5′-UTRs to modulate translation initiation. These are the G-q motifs, IRE and IRES structures. Both G-q motifs and IREs are normally negative regulators of translation and therefore could not account for the translation enhancement observed with the SNCA 5′-UTR sequence. Nevertheless, they were included in the analysis as non-canonical functions have been previously reported [37]. Bioinformatics analysis revealed three non-overlapping G-q motifs in proximal SNCA 5′-UTR. Similar to most other findings, their effect was inhibitory; separately, these motifs were not very efficient in blocking translation, however their cumulative effect mounted to 45% of translation inhibition but this was rather due to enhanced transcription or mRNA stability and not due to translation *per se*. The iron response element (IRE), an approximately 30 nt long sequence, has been identified in the 5′-UTR of a number of genes involved in iron homeostasis and is the docking site for the IRP-1 and IRP-2 RBPs. Previously, an IRE motif has been predicted for distal SNCA 5′-UTR, right before the start of the SNCA ORF, but has not yet been experimentally verified [9]. For this, using nano LC-MS/MS proteomics [38–40], over 1300 interacting proteins were pulled down from murine brain extracts incubated with a biotin-labelled SNCA 5′-UTR RNA probe (data not shown). Tens of RBPs were identified but none matched to IRP-1 or IRP-2 and no further analysis was undertaken as a result. Similarly, previous transcriptome-wide definition of IRP-1/2 bound mRNAs from murine brain and bone-marrow samples did not detect *SNCA* mRNA [19].

Several cellular mRNAs harbouring IRES elements in their long 5′-UTRs have been found to mediate cap-dependent translation better than UTR-less counterparts [30,41]. Despite efforts by the scientific community to identify sequence motif(s) responsible for IRES activity, this has not been possible and current consensus favours that the activity of these elements resides in the tertiary structure. Based on these findings, the SNCA 5′-UTR was also probed for IRES activity. Using multiple constructs and methodologies, it was confirmed that the SNCA 5′-UTR harbours an IRES element. To identify the location of the IRES element, different 5′-UTR segments were tested. From these, only the middle 1/3 part retained some activity indicating that the whole or most of the 5′-UTR is required for proper IRES activity. This finding is similar to other reported cellular IRESs, supporting the idea that the IRES motif is often spread throughout the 5′-UTR of an mRNA [31,32]. Further analysis by mutagenizing all three predicted G-q motifs in proximal SNCA 5′-UTR did not affect IRES activity, indicating that at least to some extent SNCA cap-dependent translation and IRES activity are independently regulated and that the middle segment is at the core of IRES functionality.

Currently, a number of cellular IRESes have been identified, most of which are associated with genes encoding for transcription factors, oncogenes, growth factors and apoptotic proteins. If we ignore the bias for studying such genes, this cohort suggests that IRES-dependent translation is an important regulatory mechanism to maintain cellular homeostatic processes (i) when there is need for tight and rapid control of expression independent of gene transcription and (ii) in stress conditions where cap-dependent translation is impaired. Here, several stimuli were tested for the ability to induce SNCA cap- and IRES-dependent translation. Signals such as plasma-membrane depolarization by KCl, serum deprivation, oxidative stress via ferrous ammonium, MPP^+^ and 6-OHDA were found to promote expression via the 5′-UTR. Similarly, SNCA IRES expression was induced by KCl, serum deprivation, ferrous ammonium and 6-OHDA signals. Oxidative stress, iron accumulation and serum deprivation are all known perturbations of the ageing human brain. With respect to depolarization, a switch from cap-dependent to cap-independent translation has been associated with activity-dependent plasticity [28] and a number of dendritically localized mRNAs has been shown to contain functional IRES sequences, albeit the role of these IRESs has not been determined [12]. One can only speculate on the physiological role of the IRES element on SNCA expression. SNCA is a small multi-functional chaperone, still poorly understood, involved in synaptic activity but also associated with the functions of mitochondria, ER and nucleus by associating with a great number of protein partners. Therefore, expression through IRES presumably strengthens the role of SNCA in maintaining homeostasis in broad physiological and pathophysiological conditions.

The significance of current findings relates directly to PD and other synucleinopathies. A large body of evidence has identified perturbations in growth support, iron levels and synaptic activity of degenerating neurons and SNCA is accumulated at toxic levels in these diseases. Currently, most evidence indicates that reducing SNCA protein levels may be a way to alleviate some of the symptoms. The contribution of the SNCA 5′-UTR in translation regulation and the presence of IRES indicate a novel target approach to reduce SNCA levels. Previous medicinal screens to identify natural compounds that have the capacity to efficiently inhibit SNCA expression via the 5′-UTR have yielded promising leads [42]. Despite the fact that the function of 5′-UTRs and cellular IRESs is poorly understood, it is thought that most may require auxiliary RBPs for ribosomal recruitment. These proteins act as RNA chaperones either to maintain/attain the correct tertiary structure that is required for efficient assembly of 48S ribosomal complex and/or attract canonical translation initiation factors. Consequently, identifying the RBPs that bind and modulate the SNCA 5′-UTR may serve as an additional means for altering SNCA protein synthesis in individuals afflicted with PD.

## 4. Conclusion

SNCA is a highly abundant protein whose levels of expression are critical for disease development. Multiple lines of evidence suggest that even a small increase in the normal, wild-type SNCA levels is sufficient to trigger neurodegeneration in humans, animals and *in culture* models. Understanding how SNCA levels are regulated is, therefore, an important component of any neuroprotective strategy. Towards this, we studied the role of the SNCA 5′-UTR on mRNA translation. Intriguingly, we found that inclusion of the 5′-UTR ahead of the SNCA ORF doubled protein output. Importantly, this effect was not associated with a change in mRNA levels or differential nucleocytoplasmic shuttling. Further, the presence of the 5′-UTR enhanced SNCA synthesis when cap-dependent translation via the growth signalling pathway mTORC1 was attenuated, indicating that high levels of SNCA expression are maintained during cellular stress. Analysis using different methodologies revealed that the 5′-UTR harbours an IRES element that spans most of its nucleotide sequence. IRES elements are known, among others, to sustain protein expression when cap-dependent translation is compromised. Henceforth, application of stress inducers such as prolonged plasma-membrane depolarization, serum starvation and oxidative stress in cells stimulated SNCA translation via its 5′-UTR as well as enhanced its IRES activity. Taken together, these data support the idea that the 5′-UTR mediates SNCA synthesis under diverse physiological and pathological conditions, explaining in part the abundance of SNCA in healthy neurons and its accumulation in degenerative cells.

## 5. Material and methods

### 5.1. Pharmacological compounds

PD neurotoxins MPP^+^ iodide and 6-OHDA were obtained from Sigma-Aldrich (St. Louis, MO, USA) and added to the medium to a final concentration of 80 µM and 30 µΜ, respectively. Rapamycin (Sigma-Aldrich) was used at 40–1000 µΜ (depending on cell type), as a specific mTORC1 inhibitor to reduce cap-dependent translation. Potassium chloride (40 mM) and ferrous ammonium sulfate (500 µM) (both from Sigma-Aldrich) were used to depolarize and increase iron levels, respectively. Tissue culture grade dimethylsulfoxide (DMSO, Applichem, Darmstadt, Germany) was used as a control for rapamycin and 6-OHDA treatments.

### 5.2. Antibodies

The rabbit polyclonal antibody phospho-p70 S6 kinase (Thr389, CST#9234) was purchased from Cell Signaling Technologies (Beverly, MA, USA). The mouse monoclonal antibody against SNCA (sc-20) was purchased from Santa Cruz Biotechnology (Santa Cruz, CA, USA). The chicken anti-GFP (ab13970) polyclonal antibody was obtained from Abcam (Cambridge, MA, USA). The mouse anti-GAPDH-HRP-conjugated antibody (HRP-60004) was obtained from Proteintech Europe (Manchester, UK). The mouse (CST#7076) and rabbit (CST#7074) HRP-conjugated secondary antibodies were both from Cell Signaling Technologies. The chicken (12–341) HRP-conjugated secondary antibody was obtained from Merck Millipore (Darmstadt, Germany).

### 5.3. Generation of DNA constructs

All primers used in this study are shown in the electronic supplementary material, table S1. The human SNCA, GAPDH 5′-UTRs and SNCA 5′-UTR+coding + 3′-UTR (5′-CDS-3′) or kozac + coding + 3′-UTR (kozac-CDS-3′) products were all amplified by proofreading Phusion RT-PCR (Finnzymes, Vaanta, Finland) from human SHSY-5Y total RNA extracted by the RNAzol RT reagent (MRC, Cincinnati, OH, USA). The 5′-UTR PCR products were then cloned into the single NheI restriction site of the psiCHECK2 vector (Promega, Madison, WI, USA) upstream from the *Renilla* luciferase coding sequence, while the SNCA 5′-CDS-3′ and kozac-CDS-3′ SNCA PCR products were inserted into the pcDNA3.1 (Life Technologies) vector. The SNCA bicistronic reporter plasmids were constructed by inserting the whole or fragments of human SNCA 5′-UTR into the pRF and hpRF luciferase vectors (kindly provided by Dr L Hengst and originally obtained from Dr AE Willis) [29,30]. For site-directed mutagenesis, proofreading PCR reactions with mutagenized primers were carried out using the SNCA 5′-UTR psiCHECK2 or pRF vector as a template. The PCR products were subsequently cloned into the psiCHECK2 or pRF vector. The promoter-less empty and SNCA 5′-UTR psiCHECK2 reporters were constructed by partial double digestion of the psiCHECK2 plasmid with HindIII and BamHI restriction enzymes followed by ligation of the ampicillin resistance gene obtained from pGL3-basic (Promega) plasmid. All plasmids were verified by DNA sequencing before use.

### 5.4. *In vitro* transcription

Linearized pRF plasmid was used as DNA template for the synthesis of reporter bicistronic RNAs. *In vitro* transcription was initiated from the T7 coliphage promoter of the pRF vector using T7 RNA polymerase (Takara, Otsu, Japan) and NTPs (Promega). Following transcription, DNA was digested using DNAase I (New England Biolabs, Connecticut, USA) and newly synthesized RNA was purified using acidic phenol/chloroform (Fisher BioReagents, Fair Lawn, NJ, USA) extraction.

### 5.5. Cell culture and transfection

HEK-293, SK-N-SH and Neuro-2a cells were grown in high-glucose DMEM (Sigma) supplemented with 10% fetal bovine serum (FBS) (Invitrogen) and 1% penicillin/streptomycin (Sigma). Cells were maintained at 37°C in a humidified 5% CO_2_ incubator (ThermoForma, Thermo Fisher Scientific, Waltham, MA, USA). For luciferase reporter assays, HEK-293 or SK-N-SH cells were transfected a day after plating by using Lipofectamine 2000 according to the manufacturer's instructions (Invitrogen). To ensure that transfection efficiencies and reporter load into cells were uniform across conditions, plasmid constructs and Lipofectamine 2000 reagent were prepared as a master mix before aliquoting into culture wells. Transfection efficiencies were at 70–90% at 24 h as indicated by Emerald GFP (EmGFP). For immunoblotting assays, cells were transfected at plating by using Lipofectamine 2000 according to the manufacturer's instructions. Four hours later, cells were treated with the various compounds for another 48 h.

### 5.6. Cell fractionation

Neuro-2a cells were harvested in HLB buffer (10 mM Tris pH 7.4, 10 mM NaCl, 3 mM MgCl_2_, 1 mM EGTA, 0.1% NP40 and inhibitors for RNAses (RNAseOUT, Invitrogen)) and incubated for 45 min on ice. Lysates were then centrifuged at 2500 rpm for 15 min to separate cytoplasmic (supernatant) and nuclear (pellet) fractions. After five washes with HLB buffer, nuclear pellets were further lysed in RIP buffer (25 mM Tris pH 7.4, 150 mM KCl, 5 mM EDTA, 0.5% NP40 and RNAse inhibitors) and mechanically sheared using a dounce homogenizer. Nuclear/cell membranes and debris were discarded by centrifugation at 13 000 r.p.m. for 10 min and supernatants were purified using the RNAzol RT reagent (Molecular Research Center, Cincinnati, USA).

### 5.7. Dual luciferase reporter assay

Luciferase assays were performed 48 h after transfection with the dual luciferase reporter assay system from Promega and measured on the Lumat LB9507 luminometer (Berthold Technologies, Bad Wildbad, Germany). Changes in expression of *Renilla* luciferase (target) were calculated relative to firefly luciferase (internal control) for the psiCHECK2 plasmids while the reverse applied for the pRF and hpRF plasmids.

### 5.8. Reverse transcription and PCR

RT-PCR assay was used to assess the levels of *SNCA, GAPDH* and *EmGFP* mRNAs in HEK-293 or Neuro-2a cells transfected with different plasmids or treated with different stress stimuli [43,44]. Total RNA was isolated using the RNAzol RT reagent and recovered in 100 µl DEPC-treated H_2_O. After DNAse I (NEB) treatment, 0.5 µg total RNA was reverse transcribed for 1 h at 37°C with MMLV enzyme (Life Technologies) in reaction containing the manufacturer's buffer and DTT supplemented with 0.5 mM dNTPs (Promega) and 10 µM random hexanucleotides (Amersham/GE Healthcare, Buckinghamshire, UK). For gel based PCR assays, following reverse transcription, 2 µl samples of cDNA diluted 2.5× in H_2_O 2.5× added in 10 µl PCR reactions containing 1× buffer with 2 mM MgCl_2_, 0.2 mM dNTPs, 2 pmoles gene specific primers, and 0.2 units DreamTaq polymerase (Fermentas, Burlington, Canada). cDNAs were amplified by cycling at 95°C for 25 s, followed by 25 s at 53°C, followed by 25 s at 72°C. The reaction was then completed with a 10 min extension at 72°C. Cycles were falling within the linear range of amplification for each primer pair. See electronic supplementary material, table S1 for primer sequences. The PCR products were next separated on 8% non-denaturing polyacrylamide gels. The gels were subsequently stained with SyberGold (Life Technologies) and images were captured with the Dolphin gel documentation system (Wealtech, Sparks, USA) [43,44]. The same cDNAs (1 µl) were also amplified in 20 µl PCR reactions with real-time PCR on a Light Cycler 96 instrument (Roche Applied Sciences, Penzberg, Bavaria, Germany) using 1× Rxn buffer with 1.5 mM MgCl_2_, 0.2 mM dNTPs, SYBR Green Reagent (Roche), 5 pmoles gene specific primers and 0.6 units Platinum Taq polymerase (Invitrogen). Specificity of amplification products was confirmed by their dissociation curves. Each sample was tested in triplicate and samples obtained from three to four independent experiments were used for analysis of relative expression by the comparative CT method.

### 5.9. Western blot

Immunoblotting was used to assay the protein levels of SNCA and loading controls in cell-line cultures transfected with plasmids and/or treated with pharmacological compounds [45]. Human cell lines were harvested in a lysis buffer containing 25 mM Tris pH 7.5, 150 mM NaCl, 1 mM EDTA, 1% Triton X-100, phosphatase (PhosSTOP^®^, Roche) and protease (Complete^®^, Roche) inhibitor cocktails. Cellular protein content was determined by the Bradford assay (Bio-Rad, Hercules, CA, USA). Equal amounts of cell extracts were supplemented with 6 × SDS sample buffer (375 mM Tris pH 6.8, 10% SDS, 50% glycerol, 10% β-mercaptoethanol, 0.03% bromophenol blue), boiled for 5 min and subjected to SDS-PAGE under reducing conditions on 12 or 15% polyacrylamide gels, depending on the molecular mass of the proteins under examination. After electrophoresis, the resolved proteins were transferred to Protran^®^ nitrocellulose membrane (Whatman, Kent, UK) by electroblotting. Subsequently, membranes were saturated for 1 h at room temperature in 5% non-fat milk in TBS/0.1% Tween-20 (TBS-T) buffer and incubated overnight at 4°C in 5% non-fat milk/TBS-T containing the primary antibody. All primary antibodies were used at 1 : 1000 dilution as recommended by vendors. The following day, membranes were washed in TBS-T, incubated for 1 h at room temperature in 5% non-fat milk/TBS-T containing the appropriate HRP-conjugated secondary antibody, washed in TBS-T and finally developed using the Western Lighting Plus ECL reagents (PerkinElmer, Waltham, MA, USA) according to the manufacturer's instructions. Each sample was tested in duplicate and samples obtained from three to five independent experiments were used for analysis. Densitometric analysis of immunoblotting images was performed using the image analysis software ImageJ (NIH, USA).

### 5.10. 5′-UTR sequence extraction and length analysis

The 5′-UTRs of all human, mouse, chicken and zebrafish RefSeq genes were retrieved from the Table Browser at the UCSC Genome browser website (https://genome.ucsc.edu/cgi-bin/hgTables). Analysed data are presented as median (interquartile range) using boxplot.

### 5.11. Statistical analysis

Mean values were derived from at least three experiments. The effect of different 5′-UTR constructs or treatments on SNCA expression was assessed by one-way ANOVA and LSD *post hoc* analysis. Comparisons between two groups were carried out using Student's *t*-test. Significance was defined as *p* < 0.05. Statistical analysis was performed using the SPSS software (Release 10.0.1, SPSS, Chicago, IL, USA).

## Supplementary Material

Primers used in this study

## Supplementary Material

Comparison of QGRS Mapper and translation inhibition scores between G-quantruplex motifs present in ZIC1, ADAM10, CCND3, MMP16 and SNCA 5'-UTRs.
