# Supplementary material for: Constitutive translation of human α-synuclein is mediated by the 5′-untranslated region
Source: Open Biol. 2016 Apr 20;6(4):160022. doi: 10.1098/rsob.160022 (PMC4852460; doi:10.1098/rsob.160022)
Supplement: Primers used in this study [file rsob160022supp1.pdf]

Gene	Used in	Forward primer	Reverse primer
SNCA kozac-CDS-3’	DNA3.1	ACTCGAGGCTAGCGCCGCCATGG ATGTATTCATGAAAGGACTTT	GGATCCTTCGAACATGGTCGAATAT TATTT
SNCA 5’-CDS-3’	DNA3.1	AACTCGAGGCTAGCAGGAGAAGG AGAAGGAGGAGGAC	GGATCCTTCGAACATGGTCGAATAT TATTT
GAPDH 5’-UTR	CHECK2	AGAGAATTCGCTAGC GCCTCAAGACCTTGGGCTGGGA	GAGCTAGC TGGTGTCTGAGCGATGTGGC
SNCA 5’-UTR	CHECK2	AGAGAATTCGCTAGCAGGAGAAG GAGAAGGA	GAGCTAGCGGCTAATGAATTCCTTT ACA
SNCA 5’-UTR G-q mut1	CHECK2	AGAGAATTCGCTAGCAGGAGAAG AAGAAGAAGGAGAACTAGAAGGA GGAGGACGGCGAC	GAGCTAGCGGCTAATGAATTCCTTT ACA
SNCA 5’-UTR G-q mut2	CHECK2	AGAGAATTCGCTAGCAGGAGAAG GAGAAGGAGGAGGACTAGGAGAA GAAGAACGACGACGACCAGAAGG	GAGCTAGCGGCTAATGAATTCCTTT ACA
SNCA 5’-UTR G-q mut3	CHECK2	AGAGAATTCGCTAGCAGGAGAAG GAGAAGGAGGAGGACTAGGAGG AGGAGGACGGCGACGACCAGAA GAAGCCCAAGAGAGAGGACGAGC GACCGAG	GAGCTAGCGGCTAATGAATTCCTTT ACA
SNCA 5’-UTR G-q mut1+2+3	CHECK2	AGAGAATTCGCTAGCAGGAGAAG AAGAAGAAGGAGAACTAGAAGAA GAAGAACGACGACGACCAGAAGA AGCCCAAGAGAGAGGACGAGCGA CCGAGC	GAGCTAGCGGCTAATGAATTCCTTT ACA
SNCA 3’-UTR	CHECK2	AAGCTTCTCGAGGAAATATCTTTG CTCCCA	GGATCCGCGGCCGCCATGGTCGAA TATTATTT
SNCA 5’-UTR	pRF, hpRF	AGAGAATTCGCTAGCAGGAGAAG GAGAAGGA	GGCTAGCCCATGGATTCCTTTACAC CACACT
SNCA 5’-UTR segment A	pRF	AGAGAATTCGCTAGCAGGAGAAG GAGAAGGA	GACCATGG GCGCTCGGTCGCTCGCCC
SNCA 5’-UTR segment B	pRF	GCTAGCGAATTC GCGACGCGGAAGTGAGGT	GCTAGCCCATGG TGAGGGAGCGCCCAGGAC
SNCA 5’-UTR segment C	pRF	GCTAGCGAATTCCTTGCCTTCAAG CCTTCTGCCTTT	GGCTAGCCCATGGATTCCTTTACAC CACACT
SNCA 5’-UTR segment A+B	pRF	AGAGAATTCGCTAGCAGGAGAAG GAGAAGGA	GCTAGCCCATGG TGAGGGAGCGCCCAGGAC
SNCA 5’-UTR segment B+C	pRF	GCTAGCGAATTC GCGACGCGGAAGTGAGGT	GGCTAGCCCATGGATTCCTTTACAC CACACT
Renilla	RT-PCR	ATGGCTTCCAAGGTGTAC	ACGTTCATTTGCTTGCAG
Firefly	RT-PCR	ATGGCCGATGCTAAGAAC	CACCAGGGCATACCTCTT
hs GAPDH	RT-PCR	GCACCACCAACTGCTTAG	GCCATCCACAGTCTTCTG
mmu GAPDH	RT-PCR	TCCAGTATGACTCCACTCAC	TCCTGGAAGATGGTGATGG
hs SNCA	RT-PCR	GTAGGCTCCAAAACCAAGGA	CCACTGCTCCTCCAACATTT
Emerald GFP	RT-PCR	ATGGTGAGCAAGGGCGAGGA	TACGTCGCCGTCCAGCTCGA
