# Supplementary material for: Constitutive translation of human α-synuclein is mediated by the 5′-untranslated region
Source: Open Biol. 2016 Apr 20;6(4):160022. doi: 10.1098/rsob.160022 (PMC4852460; doi:10.1098/rsob.160022)
Supplement: Comparison of QGRS Mapper and translation inhibition scores between G-quantruplex motifs present in ZIC1, ADAM10, CCND3, MMP16 and SNCA 5'-UTRs. [file rsob160022supp2.pdf]

Table S2

Gene description	Symbol	Species	Accesion Number	Motif position in 5'UTR[1]	Motif Length	QGRS Mapper Motif[2]	QGRS Mapper score[3]	Percent inhibition[4]	PubMed ID
Zic family member 1	ZIC1	human	NM_003412.3	678	23	GGGGGGGCGGGGGAGGCCGGGG	58	74%	18515550
ADAM metallopeptidase domain 10	ADAM10	human	NM_001110.3	68	18	GGGACGGGTAGGGGCGGG	42	64%	22065584
Cyclin D3	CCND3	human	NM_001760.4	65	20	GGGCGGCGGGCGGGCTGGGG	39	53%	22858673
Matrix metallopeptidase 16	MMP16	human	NM_005941.4	213	17	GGGAGGGAGGGAGAGGG	40	55%	19397366
Alpha synuclein	SNCA	human	NM_000345.3	2	20	GGAGAAGGAGAAGGAGGAGG	21	30%	
Alpha synuclein	SNCA	human	NM_000345.3	26	11	GGAGGAGGAGG	21	29%	
Alpha synuclein	SNCA	human	NM_000345.3	53	18	GGGGCCCAAGAAGGGGG	12	36%	

[1]Motif location from start of 5'-UTR.
[2]Predicted motif of G-quadruplex forming sequence according to QGRS Mapper algorithm; no overlaps included, see Kikin et al., 2006 for details.
[3]The QGRS Mapper scoring system evaluates a quadruplex forming G-rich sequences for its likelihood to form a stable G-quadruplex. Higher scoring sequences will make better candidates for G-quadruplex.
[4]Percent inhibition of translation based on G-quadruplex mutagenesis studies using the psiCHECK2 Dual-luciferase reporter assay system.
